# Small RNAs and their targets are associated with the transgenerational effects of water-deficit stress in durum wheat

**DOI:** 10.1038/s41598-021-83074-7

**Published:** 2021-02-11

**Authors:** Haipei Liu, Amanda J. Able, Jason A. Able

**Affiliations:** School of Agriculture, Food and Wine, Waite Research Institute, The University of Adelaide, Urrbrae, SA 5064 Australia

**Keywords:** Plant genetics, Plant molecular biology, Plant stress responses

## Abstract

Water-deficit stress negatively affects wheat yield and quality. Abiotic stress on parental plants during reproduction may have transgenerational effects on progeny. Here we investigated the transgenerational influence of pre-anthesis water-deficit stress by detailed analysis of the yield components, grain quality traits, and physiological traits in durum wheat. Next-generation sequencing analysis profiled the small RNA-omics, mRNA transcriptomics, and mRNA degradomics in first generation progeny. Parental water-deficit stress had positive impacts on the progeny for traits including harvest index and protein content in the less stress-tolerant variety. Small RNA-seq identified 1739 conserved and 774 novel microRNAs (miRNAs). Transcriptome-seq characterised the expression of 66,559 genes while degradome-seq profiled the miRNA-guided mRNA cleavage dynamics. Differentially expressed miRNAs and genes were identified, with significant regulatory patterns subject to trans- and inter-generational stress. Integrated analysis using three omics platforms revealed significant biological interactions between stress-responsive miRNA and targets, with transgenerational stress tolerance potentially contributed via pathways such as hormone signalling and nutrient metabolism. Our study provides the first confirmation of the transgenerational effects of water-deficit stress in durum wheat. New insights gained at the molecular level indicate that key miRNA-mRNA modules are candidates for transgenerational stress improvement.

## Introduction

Globally, the agricultural industry is tasked with the ever-growing difficulty of supplying the rapid increased demand for food production. Water deficit is a major abiotic stress that negatively affects agricultural production worldwide, particularly in rain-fed areas like the Mediterranean region. Durum wheat (*Triticum turgidum* ssp. *durum*) is an important staple food crop in Mediterranean countries, for its unique grain quality characteristics such as high protein content and amber-coloured kernels^1,2^. For cereal crops like durum wheat, water-deficit stress that occurs during reproductive growth stages can cause significant reduction in not only grain yield but also grain quality traits^3,4^. In Australia, durum wheat is mainly grown across South Australia, western Victoria and New South Wales. In the field, water-deficit stress often occurs during crop reproduction prior to anthesis, but can persist through to late grain filling^3^. In durum wheat, reproductive stage water-deficit stress significantly reduces grain yield mainly through its negative impact on grain-setting processes, such as floral initiation, fertilisation and spikelet development^3–5^. Water deficiency that continues to grain filling also affects grain quality traits including total starch content and grain protein accumulation, through its impacts on biological processes associated with dry matter accumulation such as photosynthesis and nutrient transport^3–5^.

To cope with abiotic stress, crops can induce different adaptive changes in growth and biological functions, such as morphological adjustments, physiological changes, epigenetic modifications, and the accumulation of protective metabolites and beneficial proteins^5^. Such changes assist survival in hostile environments, and can be potentially beneficial to recurring stresses. Research has shown that pre-exposure of abiotic stress in plants could induce stress memory, which facilitates the stress response system during recurring stress events, either in the current generation (in-generation), or in the following generation (trans-generation)^6–8^. Plants often exhibit different responses to repeated abiotic stress from a single stress event on many levels, including physiological, morphological or epigenetic changes. In spring barley, intense drought stress applied on the parental plants significantly affected the growth and functioning of the progeny^7^. Parental drought stress applied at the flag leaf stage enhanced the growth of thin and long roots in the progeny at low biomass cost, which was considered as an adaptive response to enhance the uptake of water and mineral nutrients^7^. Similarly, in *Polygonum persicaria*, an annual plant in the buckwheat family, drought stress applied in two previous generations significantly affected the seedling development in the third generation^6^. Seedlings originated from the stressed parents and grandparents developed longer root systems with a higher rate of extension into deep soil. Two-generational drought stress also induced greater biomass in the offspring. More importantly, the study showed that the transgenerational effects of drought stress were cumulative – two continuous generations of drought stress induced greater adaptive changes than just one generation of stress^6^. In winter oilseed rape, drought stress applied before the flowering stage had significant negative impacts on the parents in the eight studied cultivars^8^. However, the transgenerational effects on the progeny of the stressed parents was positive. The subsequent generation exhibited higher seedling vigour than the non-stressed controls due to the enhancement of metabolic processes, which could be possibly explained by stress memory mechanisms involving changes in the epi-genome^8^. Other studies have also explored the involvement of epigenetic mechanisms in transgenerational effects of abiotic stresses. For example, in rice, multi-generations of drought stress has improved the adaptability of offspring to recurring stress via epimutation accumulation^9–12^. Drought-induced DNA methylation patterns were maintained across generations, where genes related to these multi-generational epimutations participate in stress-responsive pathways with direct effects on morphological and physiological traits such as antioxidant capacity. The research suggests that epigenetic changes induced by transgenerational stress may provide a new approach to enhance stress tolerance in breeding.

Small RNAs (sRNAs) are epigenetic regulators with critical functions in plant growth, reproductive development, and abiotic stress responses^13^. Small RNAs, mainly microRNAs (miRNAs) and short interfering RNAs (siRNAs), can precisely reprogram the expression of stress- or development-related genes through post-transcriptional gene silencing^14,15^. Compared with other epigenetic regulatory mechanisms, sRNAs can quickly respond to various environmental and developmental cues, and act as mobile signalling molecules to modulate the expression level of their target genes^13,16–19^. In particular, the biological functions of many miRNA families in regulating important reproductive, agronomic and physiological traits have been described in several studies^20–24^. Research has shown that plant miRNA-mediated pathways can participate in stress memory to facilitate the adaptation to recurring stress^25^. In our previous research, we discovered that in durum wheat, water-deficit and heat stress imposed on the parents during reproduction had negative effects on seed germination and seedling vigour in the progeny, but to a lesser extent in the stress-tolerant genotype^26^. Significant differences in miRNA expression was observed between stress-tolerant and stress-sensitive genotypes under the effects of transgenerational stress. The biological functions of differentially expressed miRNA modules are related with signalling pathways in stress adaptation and plant development, which could contribute to the phenotypic differences observed in the seedlings^26^. However, there has been no investigation of the miRNA-mRNA regulatory modules in progeny exposed to water-deficit stress during reproduction to study the association between gene networks and the physiological/yield traits under the effects of transgenerational water-deficit stress.

Here, we provide the first report of transgenerational effects of water-deficit stress on the physiological traits, yield performance and grain quality in durum wheat. Next-generation sequencing analysis was carried out to profile the miRNAome, transcriptome, and degradome with or without the effects of transgenerational stress. Our data provide the first evidence in *T. turgidum* on the transgenerational effects of reproductive-stage water-deficit stress. New knowledge gained at the molecular level has revealed key miRNA-mRNA regulatory modules that are potential new candidates to enhance stress tolerance across generations in molecular breeding.

## Methods

### Plant materials, stress treatment and sample collection

Two Australian durum wheat varieties, DBA Artemis and DBA Aurora, were grown in controlled glasshouse conditions as previously described^4^. Both varieties originate from South Australia with good tolerance to abiotic stresses: DBA Aurora having higher tolerance towards water-deficit stress and DBA Artemis having higher tolerance towards heat stress^4^. The seeds used for the current study were collected from the parent plants in the control group and parent plants in the water-deficit stress group from the previous year^4^. Here, four seed groups were planted: AtC (DBA Artemis seeds from control group parents), AtW (DBA Artemis seeds from water-deficit stress group parents), AuC (DBA Aurora seeds from control group parents), and AuW (DBA Aurora seeds from water-deficit stress group parents). Progeny from each parent group were treated with control or water-deficit stress again, thus totalling eight treatment groups: AtCC (DBA Artemis control group parents, progeny treated with control), AtCW (DBA Artemis control group parents, progeny treated with water-deficit stress), AtWC (DBA Artemis water-deficit stress group parents, progeny treated with control), AtWW (DBA Artemis water-deficit stress group parents, progeny treated with water-deficit stress); AuCC (DBA Aurora control group parents, progeny treated with control), AuCW (DBA Aurora control group parents, progeny treated with water-deficit stress), AuWC (DBA Aurora water-deficit stress group parents, progeny treated with control), AuWW (DBA Aurora water-deficit stress group parents, progeny treated with water-deficit stress). See Supplementary Table S1 for a summary of the treatment groups.

Plants were grown under controlled glasshouse conditions, with day/night temperature at 22 °C/12 °C (12 h photoperiod) as previously described^4^. All plants were well-watered from germination to booting. Pre-anthesis water-deficit stress was applied from the booting stage (Zadoks stage 49) until maturity, by maintaining the soil water content in pots at 6% (equal to half of the field capacity). Both the flag leaf and developing grain samples were collected from the main tiller at 5 DPA (days post-anthesis). Total RNA was extracted using the Tri reagent (Sigma-Aldrich) and treated with TURBO DNase (ThermoFisher Scientific). The concentration, quality and integrity of total RNA samples were assessed by a NanoDrop spectrophotometer, gel electrophoresis and the Agilent Bioanalyzer prior to use in next generation sequencing and qPCR. Six biological replicates were sampled for each group. Equal pooling of total RNA samples from the six biological replicates was performed prior to next-generation sequencing analysis.

### Evaluation of crop performance – physiological traits, yield components and grain quality traits

Chlorophyll content and stomatal conductance (adaxial) were measured every 5 days from flowering (0 DPA) to 40 DPA as previously described^4,27^. Briefly, chlorophyll content was measured three times along the middle part of the flag leaf with a SPAD meter, stomatal conductance was measured on the adaxial surface along the middle part of the flag leaf with a SC-1 porometer. All replications were arranged in a complete randomised block design. Eight biological replicates per group were used for chlorophyll content measurement. Four biological replicates were randomly selected from these eight for measuring stomatal conductance. At harvest, yield components including grain weight per plant, grain number per plant, biomass, harvest index, 1000-grain weight, plant height, tiller number and main spike length were determined. Ten biological replicates were used for harvest analysis. After harvest analysis, out of these ten biological replicates, four biological replicates per group were randomly chosen for destructive grain quality analysis. Wholemeal flour samples of the mature grains were prepared using an IKA A11 analytical mill^4^ for the measurement of grain quality traits. A rapid N Elementar was used to determine grain protein content (GPC) based on the Dumas method (nitrogen content multiplied by a factor of 5.7 at 11% moisture basis). Total starch content (TSC) of the grains was determined using the Total Starch Assay Kit as previously described^4^. Statistical analysis was performed using GENSTAT 20 Edn (VSN International Ltd) as described previously^4^. One-way ANOVA was used to detect significant differences between treatment groups of the same genotype, with the least significance difference (l.s.d.) value at *P* < 0.05.

### sRNA, transcriptome, and degradome sequencing

Flag leaf RNA and developing grain RNA samples from DBA Artemis were used for small RNA, transcriptome and degradome sequencing analysis (Supplementary Table S2). The sRNA libraries were prepared using the NEBNext Multiplex Small RNA Library Prep Kit and multiplex primers, as previously described^26,28,29^. sRNA-seq was performed on Illumina HiSeq 2500 at LC-Bio in Hangzhou, China. Original datasets of sRNA-seq, transcriptome-seq, and degradome-seq generated in the current study were submitted to NCBI GEO database (accession number GSE162008). Conserved and novel durum wheat miRNAs were identified using the ACGT101-miR program as previously described^26,28,29^. All miRNAs were categorised into five groups (G1-5), where G1 to G4 represent different categories of conserved miRNAs, while G5 represents novel miRNAs. For details, see Methods S1. Normalised reads count was used to represent the expression level of miRNAs in each library. Chi-square (n × n) was used to identify differentially expressed miRNAs (DEMs) across treatment groups at *P* < 0.05. Fold-change calculations were performed for paired-comparisons made between treatment groups.

The transcriptome libraries (Supplementary Table S2) were constructed using the Illumina mRNA-Seq sample preparation kit, and sequenced on Illumina NovaSeq 6000 at LC-Bio as previously described^28^. Bioinformatics analysis of transcriptome-seq was performed as previously described^28^. For details, see Methods S1. Gene expression level was normalised in FPKM (Fragments Per Kilobase Million). The edgeR package was used to identify differentially expressed genes (DEGs) at *P* value < 0.05 using chi-square (n × n). Fold-change calculations were performed for paired-comparisons made between treatment groups.

The degradome libraries (Supplementary Table S2) were prepared and sequenced on Illumina HiSeq 2500 at LC-Bio as previously described^28^. Bioinformatics analysis of degradome-seq was performed using the CleaveLand package and the ACGT101-DEG program as previously described^28^. Degradome-seq signatures were analysed to determine mRNA targets that were post-transcriptionally regulated by durum miRNAs. For details, see Methods S1. Normalised reads abundance of target genes were expressed in TPB (transcripts per billion).

### Functional annotation of genes and integrated omics analysis

Gene Ontology (GO) annotation of *T. turgidum* DEGs was performed regarding their biological processes, cellular components and molecular functions^28,29^. KEGG pathway^30–32^ enrichment analysis was conducted to determine the functional pathways that DEGs were associated with. Integrated omics analysis combining sRNA-seq, transcriptome-seq and degradome-seq datasets was performed as previously described to identify significant miRNA-mRNA pairs with antagonistic regulatory patterns^28,29^. Briefly, all miRNA-mRNA pairs were first validated as evidenced by three types of sequencing datasets (i.e. miRNA candidates can be validated via sRNA sequencing, mRNA candidates can be validated via transcriptome sequencing, and miRNA-mRNA pairing can be validated by degradome sequencing). Secondly, from all validated miRNA-mRNA pairs, the ones where both miRNA and mRNA had shown significant differential expression were identified (*P* < 0.05, subject to the progeny stress treatment or parent stress treatment factor). Thirdly, from the significantly expressed miRNA-mRNA pairs, those where miRNA and mRNA had shown antagonistic expression patterns were identified (i.e., significantly reduced miRNA expression corresponding to significantly increased mRNA expression, or significantly increased miRNA expression corresponding to significantly decreased mRNA expression). GO and KEGG pathway enrichment analysis were performed for these identified miRNA-mRNA modules.

### qPCR profiling of stress-responsive miRNAs and targets

A total of eight stress-responsive miRNAs and 12 target genes were selected for qPCR analysis in DBA Artemis and DBA Aurora. These candidates were selected for their predicted biological functions associated with stress response and plant growth, potentially contributing to the transgenerational effects. cDNA was synthesised from treated total RNA samples with the MystiCq microRNA cDNA Synthesis Mix (Sigma-Aldrich) as previously described^27,33^. qPCR analysis was carried out on a ViiA7 Real-Time PCR machine using the PowerUp SYBR Green Master Mix as previously described^27,33^. The relative gene expression level was calculated using the durum wheat GAPDH gene as the reference gene. Data was represented as mean ± SE (three biological replicates). The three biological replicates were randomly chosen from the six biological replicates used for pooling in next-generation sequencing libraries. One-way ANOVA was used to determine statistical significance across treatment groups at *P* < 0.05 with the l.s.d. value (least significant difference). Different letters (a – c) denote the statistical difference across the treatment groups. Primer sequences are included in Supplementary Table S3.

## Results

### Crop performance of two genotypes with different seed sources

**Figure 1:**
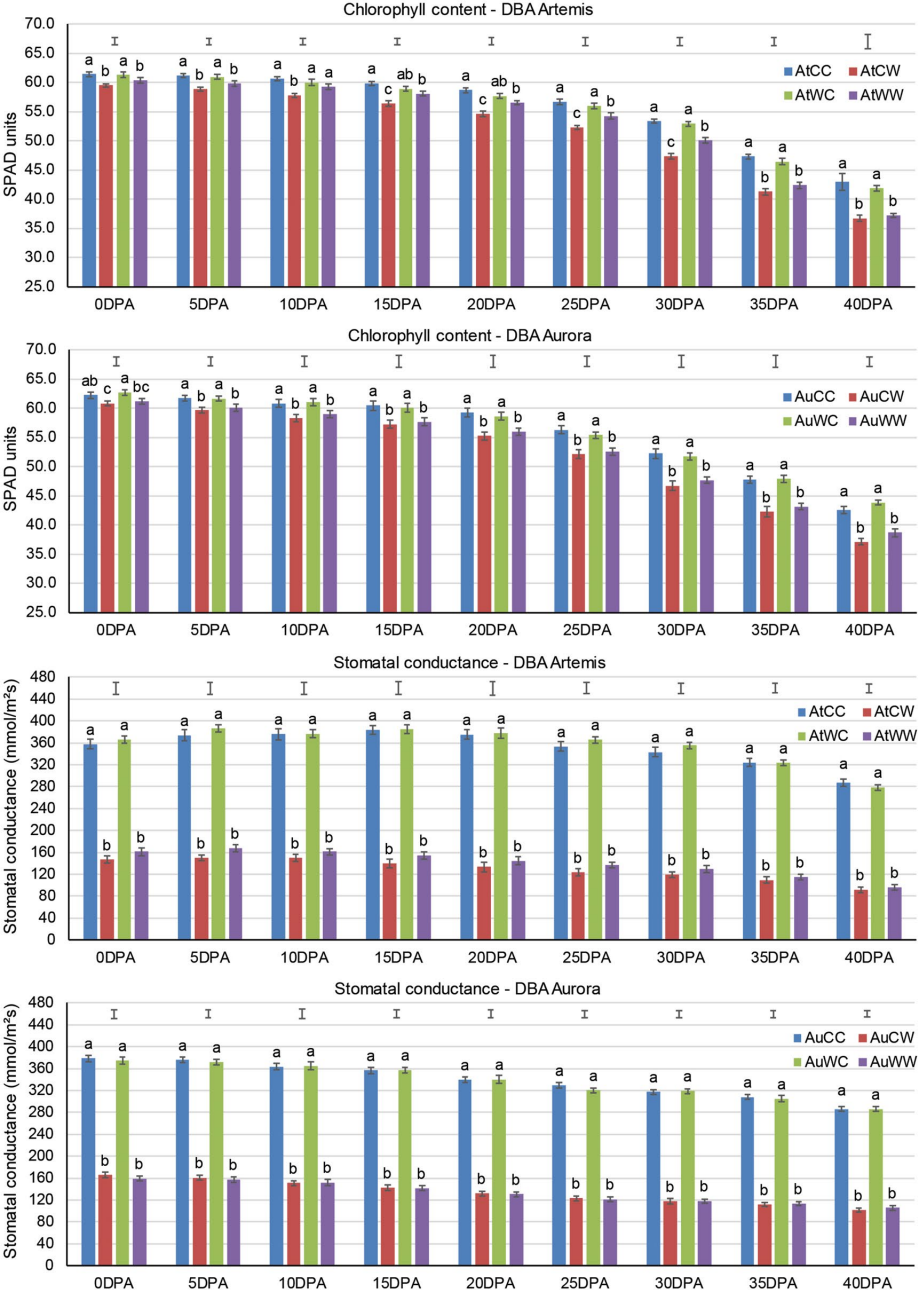
Effects of transgenerational water stress on chlorophyll content and stomatal conductance measured on the flag leaf every 5 days from flowering to 40 DPA (days post anthesis) in two durum wheat genotypes. Means ± SE for n = 8 are shown for chlorophyll content and means ± SE for n = 4 are shown for stomatal conductance. Different letters (**a**–**c**) denote statistically significant differences at the *P* < 0.05 level among treatment groups within a time-point. Capped lines show the l.s.d. values at *P* = 0.05 for comparison among treatment groups at each time-point. The treatment groups are: AtCC (DBA Artemis control group parents, progeny treated with control), AtCW (DBA Artemis control group parents, progeny treated with water-deficit stress), AtWC (DBA Artemis water-deficit stress group parents, progeny treated with control), AtWW (DBA Artemis water-deficit stress group parents, progeny treated with water-deficit stress); AuCC (DBA Aurora control group parents, progeny treated with control), AuCW (DBA Aurora control group parents, progeny treated with water-deficit stress), AuWC (DBA Aurora water-deficit stress group parents, progeny treated with control), AuWW (DBA Aurora water-deficit stress group parents, progeny treated with water-deficit stress).

Two leaf physiological traits (chlorophyll content and stomatal conductance) were measured every 5 days from flowering to 40DPA (Fig. 1). In DBA Artemis, water-deficit stress significantly reduced the chlorophyll content at all time-points in the progeny from the same parents (i.e. AtCW vs AtCC, AtWW vs AtWC), except for 10, 15 and 20 DPA in the progeny from the stressed parents, where no significant difference was found between AtWW and AtWC. Chlorophyll content between control group progeny from the control parents and the stressed parents (AtWC vs AtCC) showed no significant difference. However, under water-deficit stress, progeny from the stressed parents managed to maintain significantly higher chlorophyll content than the progeny from the control parents (AtWW vs AtCW) from 10 to 30 DPA. For DBA Aurora, water-deficit stress significantly reduced the chlorophyll content at all time-points in the progeny from the same parents (i.e. AuCW vs AuCC, AuWW vs AuWC). No significant difference was found between the control group progeny from the control parents and stressed parents (AuWC vs AuCC), and no significant difference was found between water-deficit stress treated progeny from the two parent groups (AuWW vs AuCW). For stomatal conductance, in both genotypes, water-deficit stress applied in the progeny from the same parents significantly decreased the stomatal conductance at all time-points. However, the rate of reduction under water-deficit stress compared with its control was less evident in the progeny from the stressed parents. No significant difference was found between progeny under the same progeny treatment from different parent groups.

Yield components including biomass per plant, grain weight per plant, grain number per plant, harvest index and 1000-grain weight were analysed in two durum wheat genotypes across treatment groups (Table 1). In DBA Artemis, biomass per plant, grain weight per plant, and grain number per plant exhibited the same trend – these traits significantly decreased in the progeny treated with water-deficit stress compared with the control from the same parental source (i.e. AtCW vs AtCC, AtWW vs AtWC). No significant difference was detected for progeny under the same treatment condition but originating from different parents (i.e. AtCC vs AtWC, AtCW vs AtWW). Interestingly, the harvest index of DBA Artemis progeny from the stressed parents was significantly higher than that of the progeny from the control parents, under both control and water-deficit stress conditions (i.e. AtWC vs AtCC and AtWW vs AtCW). No significant difference was detected between control and stress-treated progeny from the same parents. For 1000-grain weight, stress-treated DBA Artemis progeny from the stressed parents showed the highest value across all treatment groups. The 1000-grain weight of progeny from the control parents significantly increased under water-deficit stress (AtCW vs AtCC). For DBA Aurora, the same trend was observed for biomass per plant, grain weight per plant, and grain number per plant, where significant reductions were observed between control and stressed progeny from the same parents. No significant difference was detected for harvest index in DBA Aurora. In terms of 1000-grain weight, water-deficit stress significantly increased the 1000-grain weight in the progeny from the stressed parents (AuWW vs AtWC). No significant difference was detected between control and stressed progeny from the control parents (AuCW vs AtCC).

**Table 1:** Effect of transgenerational water stress on yield components measured at harvest for two durum wheat genotypes. Means ± SE for *n* = 10 are shown for each trait. Different letters (a, b, c) denote statistically significant differences among treatment groups within each genotype, using least significant difference (LSD) when *P* < 0.05. The treatment groups are: AtCC (DBA Artemis control group parents, progeny treated with control), AtCW (DBA Artemis control group parents, progeny treated with water-deficit stress), AtWC (DBA Artemis water-deficit stress group parents, progeny treated with control), AtWW (DBA Artemis water-deficit stress group parents, progeny treated with water-deficit stress); AuCC (DBA Aurora control group parents, progeny treated with control), AuCW (DBA Aurora control group parents, progeny treated with water-deficit stress), AuWC (DBA Aurora water-deficit stress group parents, progeny treated with control), AuWW (DBA Aurora water-deficit stress group parents, progeny treated with water-deficit stress).

	Biomass per plant (g)	Grain weight per plant (g)	Grain number per plant	Harvest index	1000-grain weight (g)
AtCC	24.51 ± 0.20 a	12.49 ± 0.13 a	292.50 ± 7.28 a	0.510 ± 0.005 b	42.93 ± 1.08 c
AtCW	20.12 ± 0.41 b	10.24 ± 0.31 b	214.80 ± 10.85 b	0.509 ± 0.010 b	48.34 ± 1.62 b
AtWC	23.52 ± 0.27 a	12.58 ± 0.24 a	269.75 ± 14.59 a	0.535 ± 0.007 a	47.34 ± 1.86 bc
AtWW	20.36 ± 0.47 b	10.95 ± 0.38 b	207.00 ± 10.84 b	0.537 ± 0.008 a	53.44 ± 1.66 a
F pr	< 0.001	< 0.001	< 0.001	0.021	< 0.001
LSD	1.034	0.805	31.260	0.023	4.477
AuCC	22.96 ± 0.24 a	12.60 ± 0.31 a	247.10 ± 9.08 a	0.549 ± 0.010	51.24 ± 0.83 ab
AuCW	19.94 ± 0.60 b	11.04 ± 0.39 b	203.90 ± 13.88 b	0.554 ± 0.012	55.42 ± 2.20 a
AuWC	23.23 ± 0.40 a	12.92 ± 0.39 a	264.20 ± 10.57 a	0.555 ± 0.010	49.16 ± 1.08 b
AuWW	19.17 ± 0.32 b	10.39 ± 0.41 b	190.60 ± 8.93 b	0.541 ± 0.015	55.03 ± 1.95 a
F pr	< 0.001	< 0.001	< 0.001	0.833	0.025
LSD	1.19	1.083	30.98	n.a	4.645

Morphological traits were evaluated at maturity for two durum wheat genotypes across treatment groups (Table 2). In DBA Artemis, plant height was significantly reduced under water-deficit stress in the progeny from the control parents, but did not have significant impact on the progeny from the stressed parents. However, both control and stressed progeny from the stressed parents exhibited significantly lower plant height compared with the control progeny from the control parents. Interestingly, in DBA Artemis, the control progeny from the stressed parents had the highest tiller number and fertile tiller number among all groups. Both tiller number and fertile tiller number were significantly reduced under water-deficit stress for the progeny from the stressed parents (AtWW vs AtWC), but showed no significant difference to that of both control and stressed progeny from the control group (AtWW vs AtCC, AtWW vs AtCW). No significant difference was detected for main spike length across treatment groups for both genotypes. For DBA Aurora, there was also no significant difference detected for plant height. Tiller number and fertile tiller number exhibited the same trend where significant reductions were observed between control and stressed progeny from the same parents.

**Table 2:** Effect of transgenerational water stress on morphological traits and grain quality traits measured at harvest for two durum wheat genotypes. Means ± SE for *n* = 10 are shown for morphological traits. Means ± SE for *n* = 4 are shown for grain quality traits. Different letters (a, b, c) denote statistically significant differences among treatment groups within each genotype, using least significant difference (LSD) when *P* < 0.05. The treatment groups are: AtCC (DBA Artemis control group parents, progeny treated with control), AtCW (DBA Artemis control group parents, progeny treated with water-deficit stress), AtWC (DBA Artemis water-deficit stress group parents, progeny treated with control), AtWW (DBA Artemis water-deficit stress group parents, progeny treated with water-deficit stress); AuCC (DBA Aurora control group parents, progeny treated with control), AuCW (DBA Aurora control group parents, progeny treated with water-deficit stress), AuWC (DBA Aurora water-deficit stress group parents, progeny treated with control), AuWW (DBA Aurora water-deficit stress group parents, progeny treated with water-deficit stress).

	Plant height (cm)	Tiller number	Fertile tiller number	Main spike length (cm)	Grain protein content (GPC%)	Total starch content (TSC%)	Flour yellowness (*b)
AtCC	74.37 ± 1.24 a	6.10 ± 0.31 b	6.00 ± 0.26 b	9.27 ± 0.13	11.55 ± 0.13 c	61.14 ± 0.88	22.74 ± 0.39 a
AtCW	69.25 ± 0.76 b	4.90 ± 0.23 c	4.80 ± 0.20 c	9.18 ± 0.09	14.83 ± 1.05 ab	57.76 ± 1.27	21.46 ± 0.25 b
AtWC	66.83 ± 1.28 bc	7.25 ± 0.31 a	7.00 ± 0.38 a	8.94 ± 0.25	13.05 ± 0.32 bc	59.13 ± 1.30	23.22 ± 0.26 a
AtWW	64.30 ± 1.15 c	5.78 ± 0.40 bc	5.33 ± 0.37 bc	8.61 ± 0.28	15.21 ± 0.77 a	57.17 ± 1.02	22.78 ± 0.28 a
F pr	< 0.001	< 0.001	< 0.001	0.091	0.008	0.116	0.008
LSD	3.209	0.918	0.869	n.a	2.071	n.a	0.919
AuCC	72.11 ± 1.61	7.00 ± 0.42 a	6.80 ± 0.42 a	8.08 ± 0.17	12.99 ± 0.55 bc	55.73 ± 0.89	21.16 ± 0.23 a
AuCW	69.97 ± 0.84	4.90 ± 0.35 b	4.60 ± 0.40 b	8.40 ± 0.20	15.39 ± 1.28 a	53.72 ± 2.66	20.09 ± 0.22 b
AuWC	71.48 ± 1.11	6.20 ± 0.44 a	6.10 ± 0.41 a	8.47 ± 0.15	12.25 ± 0.20 c	53.78 ± 1.59	20.54 ± 0.37 ab
AuWW	73.07 ± 1.05	4.90 ± 0.23 b	4.80 ± 0.20 b	8.08 ± 0.17	15.08 ± 0.61 ab	51.82 ± 2.04	19.88 ± 0.22 b
F pr	0.322	< 0.001	< 0.001	0.258	0.033	0.571	0.025
LSD	n.a	1.062	1.053	n.a	2.357	n.a	0.827

Three grain traits (GPC, TSC and colour b*) were measured at harvest (Table 2). In DBA Artemis, water-deficit stress significantly increased the GPC of progeny from the same parents (AtCW vs AtCC, AtWW vs AtWC). The GPC of the DBA Artemis progeny from stressed parents seemed to have a higher GPC compared with the progeny from the control parents under the same treatment condition (AtWC vs AtCC, AtWW vs AtCW), but the difference was not significant. For DBA Aurora, water-deficit stress also significantly increased the GPC of progeny from the same parents (AuCW vs AuCC, AuWW vs AuWC). No significant difference was detected for TSC across treatment groups for both genotypes. In DBA Artemis, flour colour (yellowness b*) was significantly decreased under water-deficit for progeny from the control parents. For progeny from the stressed parents, the flour colour was not affected by stress. The same trend was also observed in DBA Aurora, where water-deficit stress only affected the colour b* of the progeny from the control parents.

### sRNA sequencing and differentially expressed miRNAs (DEMs)

DBA Artemis samples were used for sRNA, transcriptome and degradome sequencing, based on the improvement of selected traits in DBA Artemis under the effects of transgenerational stress. A total of 90.30 million raw reads were generated for eight sRNA libraries (Supplementary Table S2). Over 69.19 million valid sRNA reads were obtained after reads filtering. From the eight sRNA libraries, a total of 1739 MIR-miRNA entries were identified (Supplementary Table S4), considering both different MIR gene origins and different mature miRNA products. Of these, 965 were conserved miRNAs from 52 MIR families registered in the miRBase, and 774 were novel miRNAs (unique to *T. turgidum*). Venn diagrams (Supplementary Fig. S1a) illustrate the distribution of miRNA numbers between different biological groups. The biological group with the highest number of unique miRNAs was AtCW, in both the flag leaf tissue and the developing grain tissue. Fig. S1b shows the conservation profile of the identified durum wheat miRNAs among the reference plant species. Expectedly, the highest conservation was found in bread wheat (*Triticum aestivum*).

Differentially expressed miRNAs (DEMs) were identified based on the normalised reads count. For a summary of the number of DEMs subject to different factors, see Supplementary Table S5. To study the effects of progeny stress treatment, paired-comparisons were made between AtCC and AtCW, AtWC and AtWW (Supplementary Table S6). For progeny from the control parents, 86 miRNAs showed significant differential expression (*P* < 0.05 and |log2foldchange|> 1) between the control and water-deficit stress group in the flag leaf (AtCW_L vs AtCC_L). In the developing grains, 571 miRNAs showed significant differential expression (*P* < 0.05 and |log2foldchange|> 1) between the control and water-deficit stress group (AtCW_G vs AtCC_G). For progeny from the stressed parents, 234 miRNAs showed significant differential expression (*P* < 0.05 and |log2foldchange|> 1) between the control and water-deficit stress group in the flag leaf (AtWW_L vs AtWC_L). In the developing grains, 456 miRNAs showed significant differential expression (*P* < 0.05 and |log2foldchange|> 1) (AtWW_G vs AtWC_G). It appears that leaf miRNAs were more responsive to water-deficit stress in the progeny from the stressed parents (higher number of DEMs compared with progeny from the control parents), while grain miRNAs were less responsive in the progeny from the stressed parents (lower number of DEMs compared with progeny from the control parents) (Supplementary Table S5).

To study the transgenerational effects of parental treatment, paired-comparisons were made between AtCC and AtWC, AtCW and AtWW (Supplementary Table S7). For progeny grown under the control conditions, 223 miRNAs showed significant differential expression (*P* < 0.05 and |log2foldchange|> 1) in the flag leaf between different parental sources (AtWC_L vs AtCC_L). In the developing grains (AtWC_G vs AtCC_G), 562 miRNAs showed significant differential expression (*P* < 0.05 and |log2foldchange|> 1). For progeny grown under water-deficit stress, 317 miRNAs showed significant differential expression (*P* < 0.05 and |log2foldchange|> 1) in the flag leaf between different parental sources (AtWW_L vs AtCW_L). In the developing grains (AtWW_G vs AtCW_G), 770 miRNAs showed significant differential expression (*P* < 0.05 and |log2foldchange|> 1). It appears that a higher number of miRNAs were affected by the transgenerational effects when the progeny were grown under water-deficit stress (Supplementary Table S5).

To study DEMs with tissue-specific patterns, paired comparisons were made between the flag leaf and developing grain samples (Supplementary Table S8). For progeny from the control parents and treated with the control conditions, 1067 miRNAs showed significant differential expression (*P* < 0.05 and |log2foldchange|> 1) between the flag leaf and the developing grain (AtCC_L vs AtCC_G). For progeny from the control parents but treated with water-deficit stress, 923 miRNAs showed significant differential expression (*P* < 0.05 and |log2foldchange|> 1, AtCW_L vs AtCW_G). For progeny from stressed parents treated with the control conditions, 1168 miRNAs showed significant differential expression (*P* < 0.05 and |log2foldchange|> 1) between the flag leaf and the developing grain tissue (AtWC_L vs AtWC_G). While finally, for progeny from stressed parents but treated with water-deficit stress, 1074 miRNAs showed significant differential expression (*P* < 0.05 and |log2foldchange|> 1, AtWW_L vs AtWW_G). It appears that a higher number of miRNAs had tissue-specific expression in the progeny from the stressed parents (Supplementary Table S5).

### Transcriptome sequencing, degradome sequencing, and bioinformatics

Sequencing of eight transcriptome libraries generated over 697.9 million valid reads (Supplementary Table S2). A total of 66,559 genes (Supplementary Table S9) were identified, and the mean abundance of genes ranged from 10.38 to 12.00 FPKM in the eight libraries.

**Figure 2:**
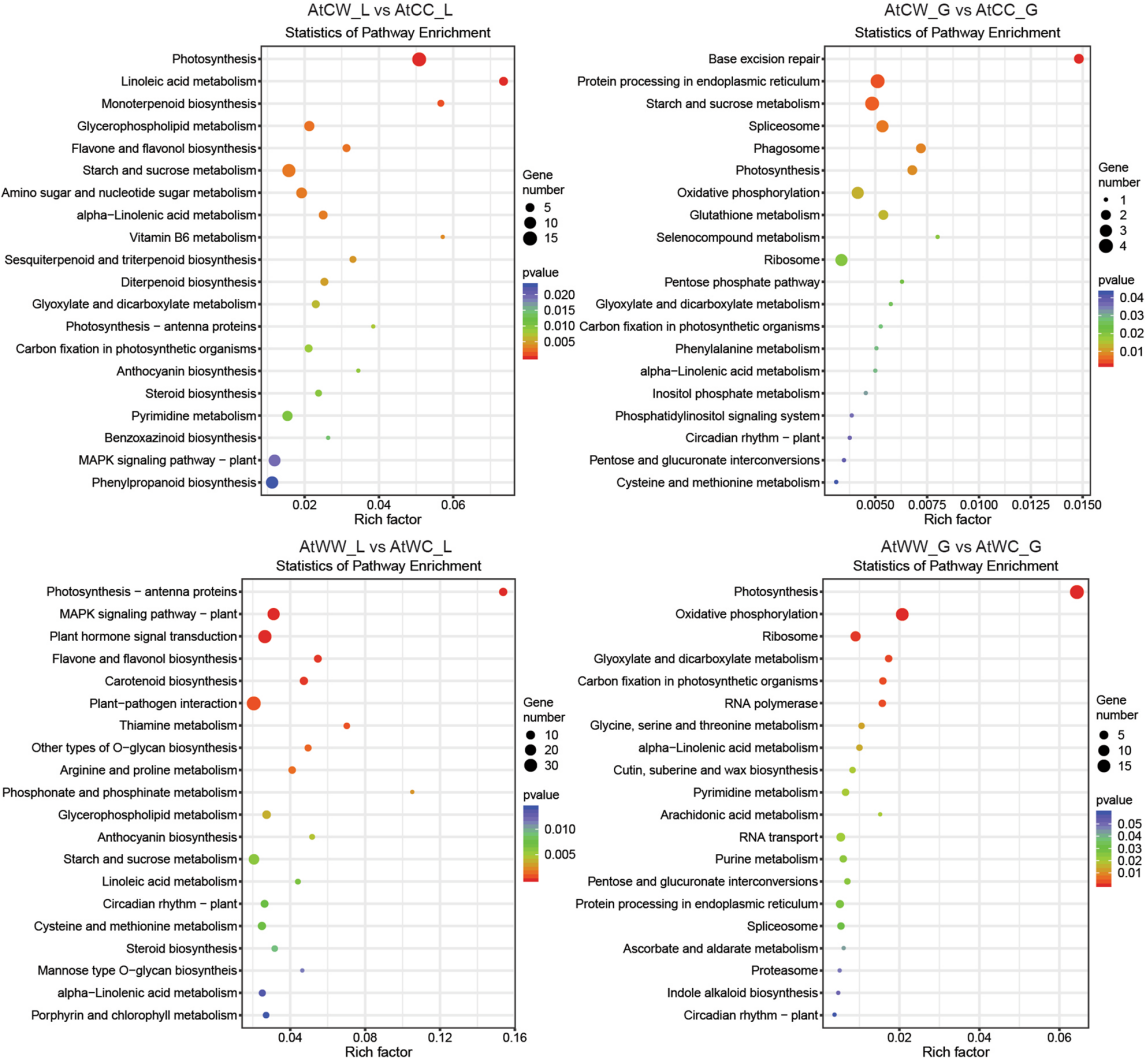
KEGG pathway enrichment analysis of stress-responsive genes in the progeny from the control parents and water-deficit stressed parents. The treatment groups are: AtCC (DBA Artemis control group parents, progeny treated with control), AtCW (DBA Artemis control group parents, progeny treated with water-deficit stress), AtWC (DBA Artemis water-deficit stress group parents, progeny treated with control), AtWW (DBA Artemis water-deficit stress group parents, progeny treated with water-deficit stress). _L represents flag leaf libraries, _G represents developing grain libraries. The rich factor represents the degree of enrichment, calculated based on the proportion of stress-responsive genes within all the genes under a specific KEGG pathway.

Differentially expressed genes (DEGs) were identified based on their normalised reads count. To study the effects of the progeny stress treatment, paired-comparisons were made between AtCC and AtCW, AtWC and AtWW (Supplementary Table S10). For progeny from the control parents, 463 genes showed significant differential expression (*P* < 0.05 and |log2foldchange|> 1) between the control and water-deficit treatment group in the flag leaf (AtCW_L vs AtCC_L). In the developing grains, 77 genes showed significant differential expression (*P* < 0.05 and |log2foldchange|> 1, AtCW_G vs AtCC_G). For progeny from the stressed parents, 727 genes showed significant differential expression (*P* < 0.05 and |log2foldchange|> 1) between the control and water-deficit treatment group in the flag leaf (AtWW_L vs AtWC_L). In the developing grains, 204 genes showed significant differential expression (*P* < 0.05 and |log2foldchange|> 1, AtWW_G vs AtWC_G). It appears that in both leaf and developing grain tissues, there were a higher number of DEGs responsive to water-deficit stress in the progeny from the stressed parents (Supplementary Table S5). Further KEGG pathway enrichment analysis provided information on the biological functions of these stress-responsive DEGs (Fig. 2). Some pathways were triggered under watered-deficit stress in both progeny from the control and the stressed parents, while some pathways are specific to progeny from the stressed parents. For example, in the flag leaf tissue, KEGG pathways such as starch and sucrose metabolism and MAPK signalling pathway − plant were enriched in progeny from both the control and stressed parents (AtCW_L vs AtCC_L; AtWW_L vs AtWC_L). Pathways like carotenoid biosynthesis and porphyrin and chlorophyll metabolism were only enriched in progeny from the stressed parents (AtWW_L vs AtWC_L).

To study the transgenerational effects of parental treatment, paired-comparisons were made between AtCC and AtWC, AtCW and AtWW (Supplementary Table S11). For progeny under the control conditions, 1057 genes showed significant differential expression (*P* < 0.05 and |log2foldchange|> 1) in the flag leaf due to water-deficit stress imposed on the parents (AtWC_L vs AtCC_L). In the developing grains, 534 genes showed significant differential expression (*P* < 0.05 and |log2foldchange|> 1, AtWC_G vs AtCC_G). For progeny under water-deficit stress, 881 genes showed significant differential expression (*P* < 0.05 and |log2foldchange|> 1) in the flag leaf due to water-deficit stress imposed on the parents (AtWW_L vs AtCW_L). In the developing grains, 532 genes showed significant differential expression (*P* < 0.05 and |log2foldchange|> 1, AtWW_G vs AtCW_G). Opposite of the pattern observed for miRNAs, there were a lower number of DEGs affected by the transgenerational effects when the progeny were grown under water-deficit stress (Supplementary Table S5).

To study DEGs with tissue-specific patterns, paired comparisons were made between flag leaf and developing grain samples (Supplementary Table S12). For progeny from the control parents treated with the control conditions, 9316 genes showed significant differential expression (*P* < 0.05 and |log2foldchange|> 1) between the flag leaf and the developing grain tissue (AtCC_L vs AtCC_G). For progeny from control parents treated with water-deficit stress, 9363 genes showed significant differential expression (*P* < 0.05 and |log2foldchange|> 1, AtCW_L vs AtCW_G). For progeny from the stressed parents treated with the control conditions, 9649 genes showed significant differential expression (*P* < 0.05 and |log2foldchange|> 1) between the flag leaf and the developing grain tissue (AtWC_L vs AtWC_G. For progeny from the stressed parents treated with water-deficit stress, 9494 genes showed significant differential expression (*P* < 0.05 and |log2foldchange|> 1, AtWW_L vs AtWW_G). Similarly, this pattern was observed for miRNAs, where more DEGs showed tissue-specific expression in the progeny from the stressed parents (Supplementary Table S5).

Degradome sequencing generated over 676.58 million raw reads from the eight degradome libraries (Supplementary Table S2). For each degradome library, the number of reads aligned to mRNA transcripts ranged from 146,426 to 167,119. Degradome signatures consistent with miRNA-guided cleavage events within the mRNA transcripts were analysed. A total of 218,179 and 222,077 miRNA-guided target sites were identified in the flag leaf and developing grain libraries, respectively (Supplementary Tables S13 & S14). Degradomics data, with pairing information (paired and unpaired sites, miRNA-mRNA complementary sequence) between durum miRNAs and their targets, is listed in Supplementary Tables S13 and S14.

### Integrated omics analysis of miRNA-mRNA regulatory modules

**Figure 3:**
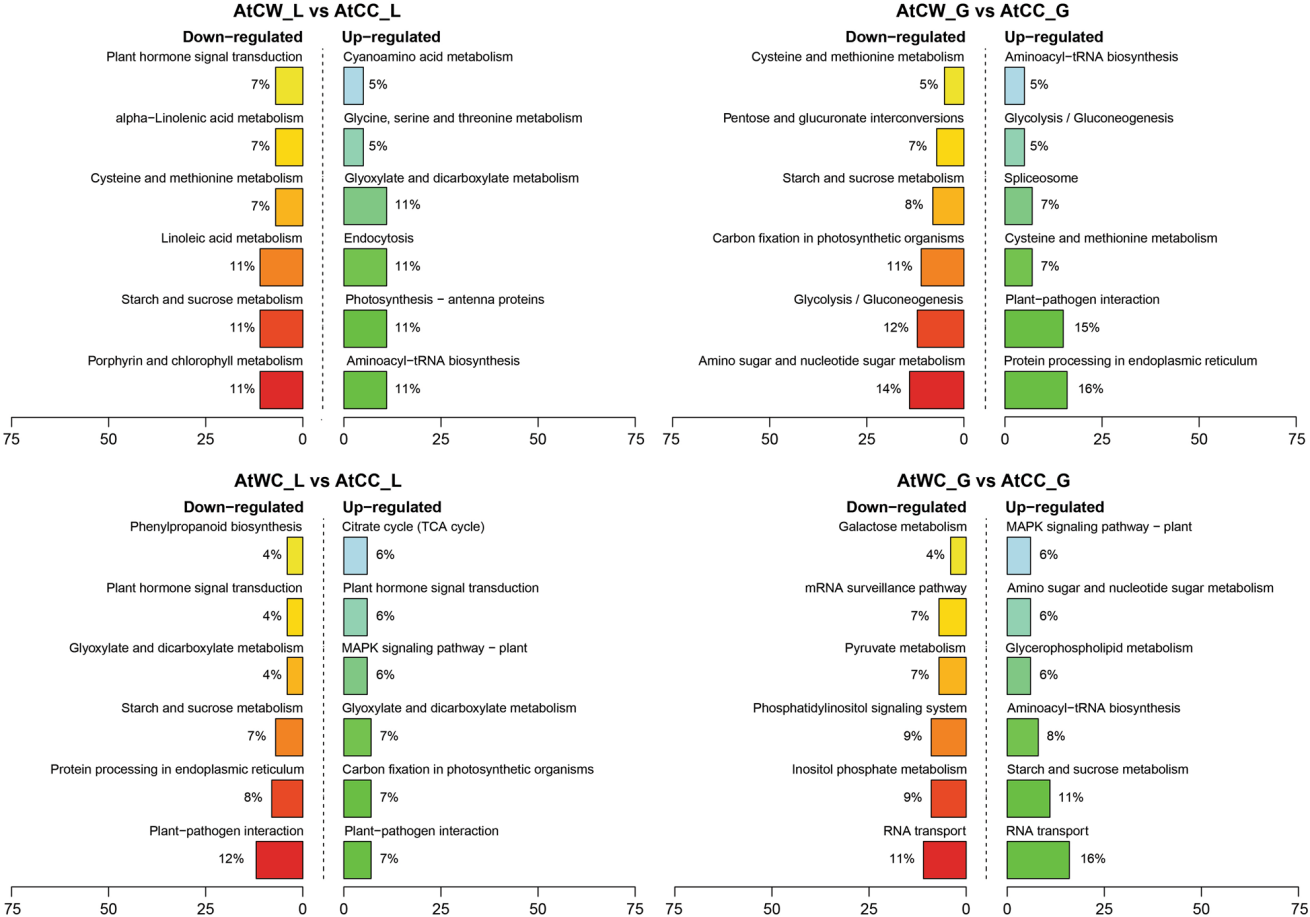
KEGG pathway classification of stress-responsive miRNA-miRNA regulatory modules. Down-regulated, mRNA targets were less abundant in the water-deficit stress treated progeny. Up-regulated, mRNA targets were more abundant in the water-deficit stress treated progeny. The treatment groups are: AtCC (DBA Artemis control group parents, progeny treated with control), AtCW (DBA Artemis control group parents, progeny treated with water-deficit stress), AtWC (DBA Artemis water-deficit stress group parents, progeny treated with control), AtWW (DBA Artemis water-deficit stress group parents, progeny treated with water-deficit stress). _L represents flag leaf libraries, _G represents developing grain libraries.

With data from the three sequencing platforms, integrated omic analysis was performed to identify miRNA-mRNA modules with significant antagonistic regulatory patterns, based on the gene silencing effects of miRNAs (Supplementary Table S15). First, subject to the progeny stress treatment factor, paired-comparisons were made between AtCC and AtCW, AtWC and AtWW to identify miRNA-mRNA pairs with significant antagonistic regulatory patterns (i.e. significant down-regulated miRNA expression, matching significant up-regulated mRNA target expression; or significant up-regulated miRNA expression matching significant down-regulated mRNA target expression) (Supplementary Table S15). For the progeny from control parents, 100 miRNA-mRNA pairs showed antagonistic regulatory patterns in the flag leaf tissue (AtCW_L vs AtCC_L); in the developing grains, 359 miRNA-mRNA pairs were found (AtCW_G vs AtCC_G). For the progeny from the stressed parents, 400 miRNA-mRNA pairs showed antagonistic regulatory patterns (AtWW_L vs AtWC_L); in the developing grains, 344 miRNA-mRNA pairs were found (AtWW_G vs AtWC_G). Notably, in the flag leaf, the progeny from the stressed parents had a lot more (four times) stress-responsive miRNA-mRNA regulatory pairs than the progeny from the control parents (Supplementary Table S15). Figure 3 shows the top KEGG pathways enriched among down-regulated and up-regulated miRNA-target pairs in the progeny treated with water-deficit stress. In the progeny from the control parents, the top four KEGG pathways where genes were up-regulated in the flag leaf tissue were aminoacyl-tRNA biosynthesis, photosynthesis—antenna proteins, endocytosis, and glyoxylate and dicarboxylate metabolism; while in the developing grain the top enriched KEGG pathway for up-regulated genes was protein processing in endoplasmic reticulum (Fig. 3). In the progeny from the stressed parents where genes were up-regulated in the flag leaf, the top three KEGG pathways were glyoxylate and dicarboxylate metabolism, carbon fixation in photosynthetic organisms and plant-pathogen interaction; while in the developing grain the top enriched KEGG pathway for up-regulated genes was RNA transport (Fig. 3).

To identify miRNA-mRNA pairs responsive to the transgenerational effects of parental treatment, comparisons were made between AtCC and AtWC, AtCW and AtWW (Supplementary Table S15). For the progeny under the control conditions, 424 miRNA-mRNA pairs showed antagonistic regulatory patterns in the flag leaf in response to stress imposed on the parents (AtWC_L vs AtCC_L); in the developing grains, 495 miRNA-mRNA pairs were found (AtWC_G vs AtCC_G). For the progeny under water-deficit stress, 365 miRNA-mRNA pairs showed antagonistic regulatory patterns in the flag leaf in response to stress imposed on the parents (AtWW_L vs AtCW_L); in the developing grains, 770 miRNA-mRNA pairs were found (AtWW_G vs AtCW_G).

### qPCR analysis of stress-responsive miRNAs and target genes

**Figure 4:**
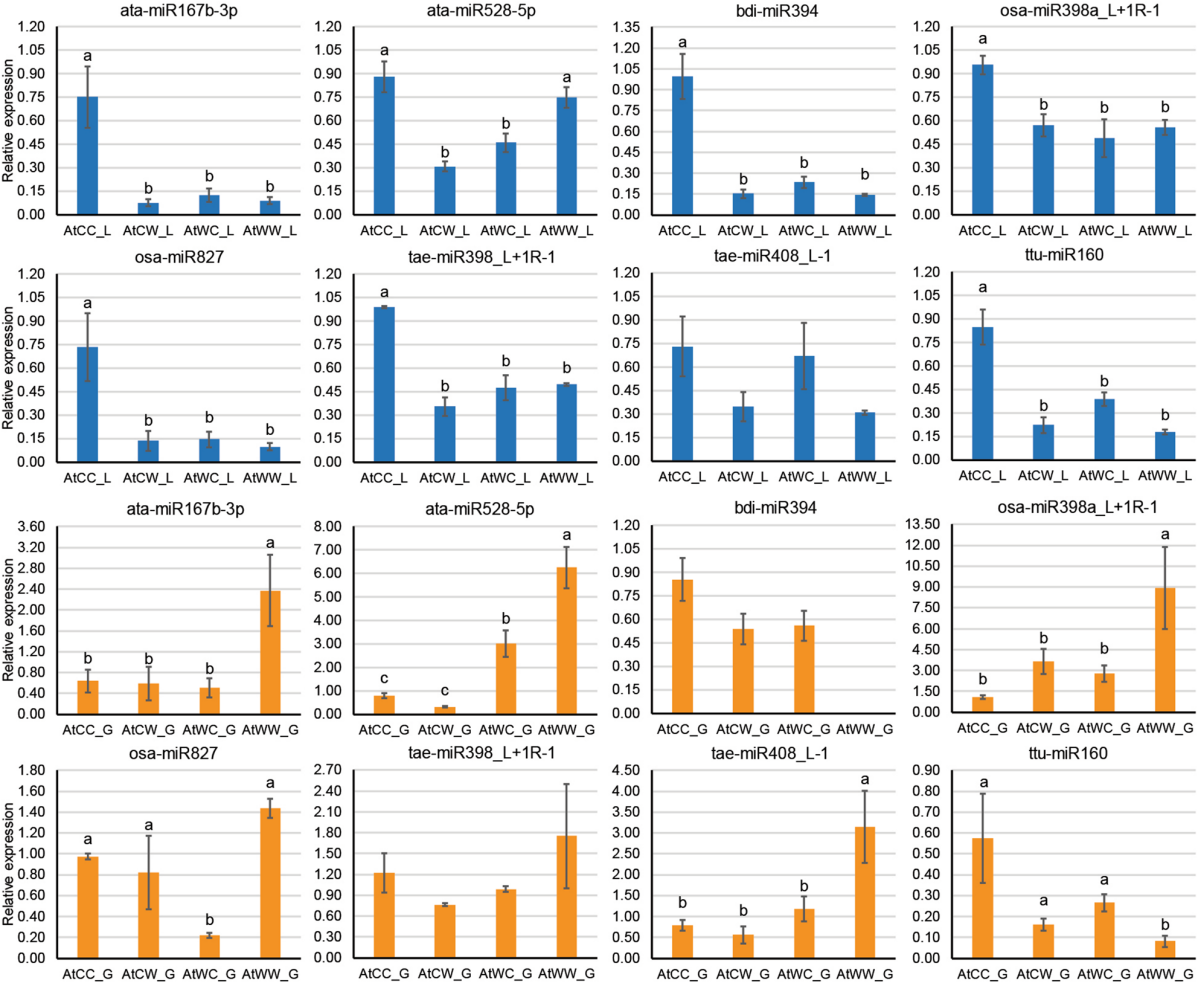
qPCR analysis of eight stress-responsive miRNAs in the flag leaf tissue (denoted with _L, highlighted in blue) and in the developing grains (denoted with _G, highlighted in orange) of DBA Artemis. Relative miRNA expression was calculated using GAPDH as the housekeeping gene. Data are presented as the mean ± standard error (SE) (n = 3). One-way ANOVA was used to determine statistical significance across treatment groups at *P* < 0.05 with the l.s.d. value (least significant difference). Different letters (**a**–**c**) denote the statistical difference across the treatment groups. The treatment groups are: AtCC (DBA Artemis control group parents, progeny treated with control), AtCW (DBA Artemis control group parents, progeny treated with water-deficit stress), AtWC (DBA Artemis water-deficit stress group parents, progeny treated with control), AtWW (DBA Artemis water-deficit stress group parents, progeny treated with water-deficit stress).

**Figure 5:**
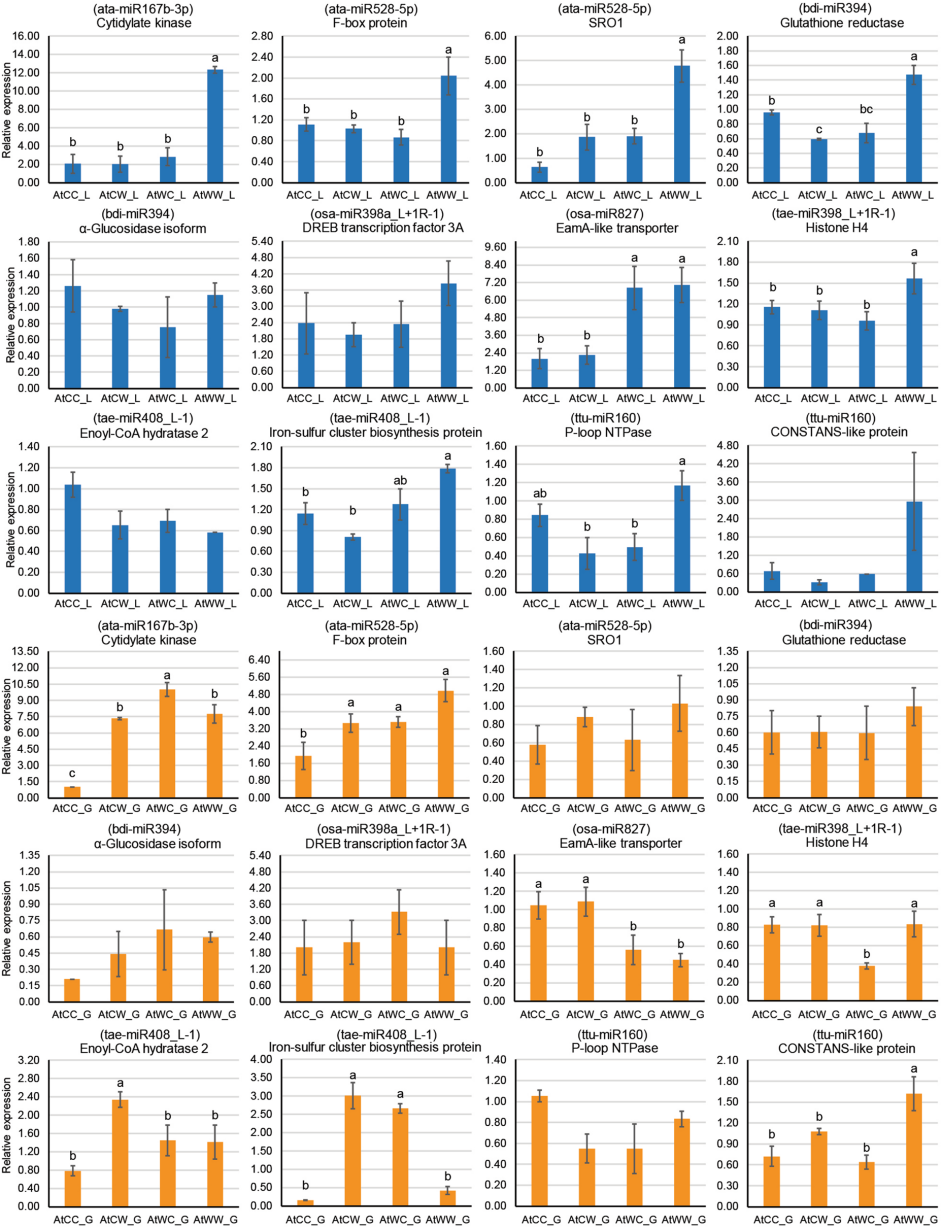
qPCR analysis of 12 stress-responsive target genes in the flag leaf tissue (denoted with _L, highlighted in blue) and in the developing grains (denoted with _G, highlighted in orange) of DBA Artemis. The miRNA targeting the gene is shown in brackets. Relative gene expression was calculated using GAPDH as the housekeeping gene. Data are represented as the mean ± standard error (SE) (n = 3). One-way ANOVA was used to determine statistical significance across treatment groups at *P* < 0.05 with the l.s.d. value (least significant difference). Different letters (**a**–**c**) denote the statistical difference across the treatment groups. The treatment groups are: AtCC (DBA Artemis control group parents, progeny treated with control), AtCW (DBA Artemis control group parents, progeny treated with water-deficit stress), AtWC (DBA Artemis water-deficit stress group parents, progeny treated with control), AtWW (DBA Artemis water-deficit stress group parents, progeny treated with water-deficit stress). SRO1, poly [ADP-ribose] polymerase SRO1 gene.

qPCR analysis was performed on eight stress-responsive miRNAs (Fig. 4 for DBA Artemis, Supplementary Fig. S2 for DBA Aurora) and 12 stress-responsive targets (Fig. 5 for DBA Artemis, Supplementary Fig. S3 for DBA Aurora). Significant differential expression across treatment groups were observed for certain miRNAs and target mRNAs, with specific patterns subject to the progeny or parental treatment factor. In DBA Artemis, for example, in the flag leaf, some miRNAs (such as ata-miR167b-3p, bdi-miR394 and osa-miR398a_L + 1R-1) were significantly reduced under water-deficit stress in the progeny from the control parents, at the same time showing no statistical difference when compared with the progeny from the stressed parents (Fig. 4). Interestingly, in the developing grains, the expression of ata-miR167b-3p and osa-miR398a_L + 1R-1 was only significantly increased under water-deficit stress in the progeny from the stressed parents, while their expression in the control progeny from the stressed parents showed no significant difference to the progeny from the control parents (Fig. 4). Different patterns could also be observed for mRNA target genes in DBA Artemis (Fig. 5). Several genes showed the opposite responsive pattern to water-deficit stress in progeny with different parental sources. For example, in the developing grains, the expression of a cytidylate kinase (target of ata-miR167b-3p) and a member of the iron-sulfur cluster biosynthesis protein family (target of tae-miR408_L-1) were significantly increased under water-deficit stress in the progeny from the control parents, but significantly reduced under stress in the progeny from the stressed parents (Fig. 5). In the flag leaf, many genes had the highest expression in the stress-treated progeny from the stress parents, with no statistical difference found across the other three treatment groups. These genes included the cytidylate kinase, a F-box protein (target of ata-miR528-5p), a poly [ADP-ribose] polymerase SRO1 (target of ata-miR528-5p) and a histone H4 gene (target of tae-miR398_L + 1R-1). Similarly, in DBA Aurora, different expression patterns can also be observed for miRNAs and their targets, depending on the progeny or parental treatment factor. For example, ttu-miR160 was significantly up-regulated under water-deficit stress in the progeny from the control parents, but was significantly down-regulated under stress in the progeny from the stressed parents (Supplementary Fig. S2). One target of tae-miR408_L-1, an enoyl-CoA hydralase gene, had significantly higher expression in the progeny from the control parents than the ones from the stressed parents (Supplementary Fig. S3).

## Discussion

Water-deficit stress induces a wide range of changes in the biological processes of crop plants, including molecular modifications, morphological changes, physiological adaptation and adjustments to reproductive processes^3,5,34–36^. Exposure of abiotic stress (short or prolonged) is known to have transgenerational effects in the subsequent generation(s). The underlying mechanisms relies on stress memory, which can be defined as the genetic, epigenetic, structural and/or biochemical modifications that can affect how a plant responds to a subsequent stress episode^37,38^. Most often, the stress memory, or imprint is beneficial to the plant, which makes it more tolerant (or more sensitive in certain cases) to future stress events in the same or subsequent generation(s)^7–9,39^. In this context, stress acclimation training presents new opportunities for agricultural researchers and breeding programs to develop novel climate-smart crop varieties. To achieve this, we must first fully understand the consequences of transgenerational stress in the targeted crop germplasm.

In the present study, reproductive-stage water-deficit stress imposed on the parents of Australian durum wheat varieties showed significant impacts at the physiological level. In crops, photosynthesis plays a central role in energy metabolism, being a key physiological trait for assessing plant fitness under adverse conditions^40,41^. In general, water-deficit stress inhibits the photosynthesis process in the leaves, mainly due to oxidative damage in the photosynthesis apparatus, which can be reflected by significantly reduced chlorophyll content^40,41^. Here, during early to mid-stages of grain fill (10–30 DPA), under water-deficit stress, DBA Artemis progeny plants from the stressed parents had significantly higher chlorophyll content than those progeny originating from the control parents (Fig. 0.1). Moreover, in DBA Artemis, although progeny from both the control and stressed parents showed significantly reduced stomatal conductance under water-deficit stress, the rate of reduction compared with its control was less evident in the progeny from the stressed parents (Fig. 0.1). The results suggest that transgenerational stress had potentially beneficial impacts on the photosynthetic and the transpiration processes in the next generation. Leaf stomatal conductance controls the gas exchange and water vapour diffusion between plants and the atmosphere^42^. Under water-deficit stress, stomatal conductance plays a key role in coordinating the CO_2_ availability and cellular water relations^43,44^. Plants can benefit from stomatal closure under water-deficit stress to maintain a higher cellular water potential, but high rate of stomatal closure can significantly inhibit the CO_2_ assimilation process^3,43,44^. Therefore, less reduced stomatal closure in the progeny plants from the stressed parents can be considered as an adaptive change to allow for greater stomatal opening, which would contribute to higher cellular water turgidity and less oxidative damage to the photosynthetic apparatus, as reflected by the higher chlorophyll content. Efficient photosynthesis under water-deficit stress will have positive impacts towards successful reproductive development, which ultimately affects crop yield and grain quality^3,4^.

The positive impacts of transgenerational stress was significant on selected yield components as well as grain quality characteristics in the current study. In DBA Artemis, the harvest index significantly increased in the progeny from the stress parents compared with progeny from the control parents. Notably, when exposed to stress, progeny from the stressed parents produced the highest 1000-grain weight and grain protein content. The grains of progeny from the stressed parents also had higher flour yellowness (b*), which is a preferential grain quality trait that gives the grain its amber colour. Similarly, in bread wheat, drought stress applied at reproductive stages in the previous generation had positive impacts on the yield components and grain quality traits in the successive generation when exposed to salt stress^45^. The progeny of stressed parents performed better with respect to productive tiller number, grains per spike, grain yield and harvest index when compared to progeny of well-watered parents^45^. The transgenerational effects of water-deficit stress can be partially explained by changes in seed provisioning and nutrient composition of wheat grains. In the bread wheat study, seeds from the stressed crops had a higher percentage of protein and total soluble phenolics than the well-watered seed source^45^. Similarly, our previous research^4^ has also shown that the grain protein content of DBA Artemis was significantly increased by water-deficit stress, while the total phenolic content remains unaffected (DBA Artemis was denoted as breeding line L2 in the previous study, with the variety commercially released post-publication). Changes in the concentration of grain nutrients were mainly affected by limited water availability during reproductive development. The accumulation of stress proteins in developing wheat grains can significantly increase under water-deficit stress, which would also affect the protein–protein interaction network^46,47^. The stress-responsive miRNA-guided regulation of storage protein biosynthesis could play a key role here^29,48^. The accumulation of stress proteins, storage metabolites and compounds with antioxidant properties (such as phenolics) in maternal plants may potentially facilitate more growth-effective metabolic processes and a swift response in progeny plants upon recurring stress conditions^8,45^. The decrease of specific miRNAs under water-deficit stress can lead to higher expression of protein synthesis-related genes and increased accumulation of protein bodies in wheat grains^8,45^. Future research could further explore the association between changes in individual grain nutrients and progeny stress performance. It is also worth noting that the impacts of transgenerational stress varied depending on the genotype, and therefore might not be generalised within a crop species. In the current study, the beneficial effects of the parental stress treatment was predominantly observed in DBA Artemis, which has lower tolerance to water-deficit stress than DBA Aurora. Similarly in rice, varieties with contrasting drought resistance levels showed genotypic differences in response to successive generations of drought stress training^10^. At the molecular level, the stress-sensitive variety had a higher rate of stable transmission of DNA methylation variations across generations when compared with the stress-tolerant variety^10^. Therefore, cross-generational stress improvement could potentially be more effective in germplasm that are less tolerant to abiotic stress, but have other superior agronomic traits for breeding purposes. However, further experimental evidence using different stress-tolerant varieties should be sought. Future research could therefore include a diverse germplasm panel with different sensitivity levels to stress, and investigate both the physiological and molecular level. It is also worth noting that differences in the grain traits from the control and stressed parents could have contributed to the varied crop performance in the next generation. Our previous report had characterised the grain weight per plant, 1000-grain weight, total protein content, total starch content and phenolics content of the grains harvested from control and stressed parents^4^. Interestingly, the water-deficit stressed DBA Aurora parents produced bigger grains than the control parents (significantly higher 1000-grain weight) while no significant difference was observed in DBA Artemis. Grains from the stressed parents of both varieties had significantly higher grain protein content compared with the control. For total starch content and total phenolic content, both traits remained unaffected for DBA Artemis but decreased significantly in the stressed DBA Aurora parents. Research has shown that a difference in parent seed size and seed reserves (such as storage proteins and carbohydrates), can significantly affect the growth rate of the progeny particularly during seedling stages, thereby potentially influencing final crop performance^49–51^. Future research should further explore the associations between different parental seed traits (morphological or quality aspects) and progeny growth traits (yield, morphological or physiological) in durum wheat germplasm.

**Figure 6:**
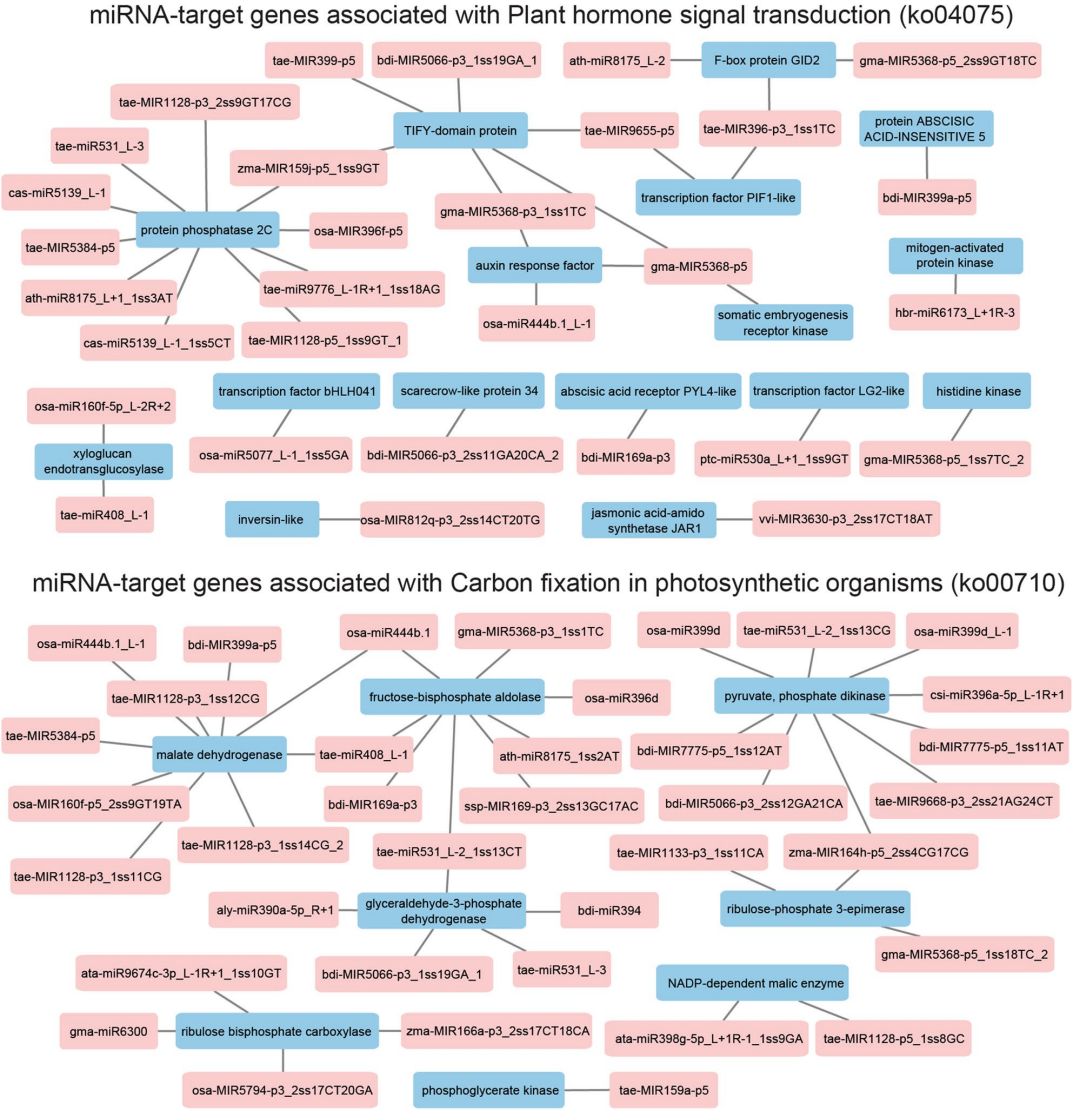
Significant miRNA-modules with antagonistic regulatory patterns, during transgenerational water stress, that are involved in KEGG (https://www.kegg.jp/kegg/kegg1.html) pathways ko04075—Plant hormone signal transduction and ko00710—Carbon fixation in photosynthetic organisms, respectively. miRNAs are shown in pink, mRNA targets are shown in blue. F-box protein GID2, an F-box subunit of the SCF E3 complex. TIFY-domain protein, protein with conserved amino acid pattern (TIF[F/Y]XG).

Cross-generation stress improvement relies on changes in the genetic and/or epigenetic regulation at the molecular level. Here, we investigated the effects of transgenerational water-deficit stress on the miRNA-guided gene regulation using three next-generation sequencing omics platforms. Overall, in the flag leaf, there were a higher number of stress-responsive DEMs and DEGs in the progeny from the stressed parents compared with the progeny from the control parents (Supplementary Table S5), suggesting that the miRNA and mRNA populations were more responsive under the effects of transgenerational stress. Specifically, miRNA-mRNA modules that have exhibited significant differential regulatory patterns between progeny with different sources have been noted. Figure 6 shows the significant miRNA-mRNA modules with antagonistic regulatory patterns (from Supplementary Table S15) that are involved in key biological pathways (e.g. ko04075—plant hormone signal transduction, and ko00710—carbon fixation in photosynthetic organisms). The functional targets in the plant hormone signal transduction pathway include genes like auxin response factor, protein phosphatase 2C, TIFY-domain proteins, and jasmonic acid-amido synthetase (Fig. 6). The functional targets in the carbon fixation in photosynthetic organisms pathway include genes like malate dehydrogenase, fructose-bisphosphate aldolase and ribulose bisphosphate carboxylase (Fig. 6). Under water-deficit stress, up-regulation of functional targets induced by lower miRNA expression could contribute to the accumulation of protective proteins and metabolites, increasing the chances of plant fitness and survival. For example, in the flag leaf tissue of DBA Artemis, a target of bdi-miR394, a glutathione reductase gene was significantly up-regulated in the stress-treated progeny from the stressed parents, and showed the highest expression level across all treatment groups (Fig. 5). Glutathione reductase plays a key role in the ascorbate–glutathione (Asc-Glu) cycle, which promotes the scavenging of reactive oxygen species (ROS). In cowpea and common bean plants^52,53^, increased glutathione reductase gene expression and protein activity has significantly contributed to the capacity of stressed plants to maintain cellular homeostasis through an enhanced antioxidant capacity. Moreover, the Asc-Glu cycle in the chloroplast can provide antioxidant protection to the photosynthetic pigments through effective detoxification of the excess ROS, which can contribute to a higher photosynthesis efficiency in the plant^40,54,55^. In bread wheat, increased expression of genes in the Asc-Glu cycle had contributed to stable photosynthetic capacity under drought stress, as shown by a high photosynthetic rate and chlorophyll content^40^. High stomatal conductance was also maintained to achieve better balance of water loss and CO_2_ assimilation^40^. Similarly in the current study, the stress-primed progeny showed higher chlorophyll content and stomatal conductance in accordance with higher expression of the glutathione reductase gene. Here, the positive transgenerational effects via miRNA regulation in durum provides a new option to enhance crop performance. In the flag leaf tissue of DBA Artemis, the expression of bdi-miR394 was significantly lower in both the control and stressed progeny from the stressed parents compared with the control progeny from the control parents, which showed no statistical difference to the stressed progeny from the control parents (Fig. 4). In this case, the results suggest that in DBA Artemis, the less stress-tolerant variety, the miR394-glutathione reductase module was primed under the transgenerational effects, and the lowered miR394 abundance allowed for rapid accumulation of glutathione reductase upon stress occurrence. This could serve the stress adaptation process via maintaining the cellular redox balance and allowing for enhanced photosynthetic performance. However, given that the glutathione reductase gene did not show any significant change in the flag leaf of DBA Aurora (Supplementary Fig. S3), this mechanism appears not to be active in DBA Aurora. For bdi-miR394, it showed higher expression in the stressed progeny from the control parents and control progeny from the stressed parents, while stressed progeny from the stress parents showed no significant difference to the control progeny from the control parents (Supplementary Fig. S2). In DBA Artemis, the ata-miR167b-3p and cytidylate kinase pair which also showed transgenerational stress-induced expression may also contribute to the photosynthetic process via the role of cytidylate kinase in energy management in the chloroplast^56^. Further studies could investigate the expression patterns of these modules across different vegetative and reproductive stages under the effects of transgenerational stress, with links to the physiological performance, to determine whether they can be used as early indicators of a broader adaptive response.

Another example of the transgenerational effects on miRNA regulation is the pair of osa-miR827 and the EamA-like transporter family. Similar to bdi-miR394, in DBA Artemis, the expression of osa-miR827 in the flag leaf was significantly lower in the progeny from the stressed parents, compared with the control progeny from the control parents (Fig. 4). Its target gene, the EamA-like transporter family member, correspondingly showed significantly higher expression in the progeny from the stressed parents (Fig. 5). However, neither osa-miR827 or the target gene exhibited any significant expression change in the flag leaf of DBA Aurora (Supplementary Figs. S2,S3). The MtN21/EamA-like transporters were first considered as nodulin proteins, but with emerging new functions in amino acid and auxin transport in non-nodulating species^57^. Studies in Arabidopsis demonstrate that members in this family are novel vacuolar facilitators of auxin transport that are required for cellular auxin homoeostasis^58,59^. So far, the MtN21/EamA-like transporters have not been functionally characterised in durum wheat. However, interestingly, the EamA-like transporter gene has been reported in several major QTL studies in cereal crops. In bread wheat, it is located underlying major QTLs for kernel weight^60^ as well as spike number per unit area^61^. The possible contribution of the EamA-like transporters towards abiotic stress tolerance has also been noted. In Egyptian spring barley^62^, a molecular marker associated with increased proline content, increased chlorophyll content, and decreased reduction in grain yield under heat stress encodes an EamA-like transporter family protein with potential positive functions towards thermo-tolerance. In chickpea, several single nucleotide polymorphisms were found in the MtN21/EamA-like transporter genes, which showed significant association with better crop yield performance under drought conditions^63^. In durum wheat, the transgenerational effects on the osa-miR827 and EamA-like transporter module could have similar positive impacts towards maintaining chlorophyll content and yield stability, as observed in DBA Artemis under water-deficit stress but not in DBA Aurora where the transgenerational effects were less pronounced. Future research could aim to determine and validate the exact function(s) of osa-miR827 and the EamA-like transporter in both stress-tolerant and -sensitive durum varieties, thereby exploring any possible relationship between auxin transport and transgenerational memory.

In conclusion, our research confirms that the reproductive-stage water-deficit stress does have transgenerational effects in durum wheat, affecting the physiological parameters, yield performance and grain quality traits in the successive generation. Epigenetic mechanisms such as miRNA-guided gene regulation provide biological links between plant phenotypes and their adaptation to environmental stress exposure. Here, inter- and trans-generational stress had a significant influence on the expression profiles of miRNAs and their protein-coding genes, of which the functions are directly linked to biological pathways in plant development and fitness. The results suggest that durum miRNA-regulated pathways could play important roles adaptation to water-deficit stress across generations, helping plants achieve a balance between stress survival and reproductive success. The key miRNA-mRNA modules identified in the study provide new entry points for epi-breeding, which could subsequently lead to the development of superior, climate-smart durum varieties.

## Supplementary information

Supplementary Legends.

Supplementary Figure 1.

Supplementary Figure 2.

Supplementary Figure 3.

Supplementary Information.

Supplementary Table 1.

Supplementary Table 2.

Supplementary Table 3.

Supplementary Table 4.

Supplementary Table 5.

Supplementary Table 6.

Supplementary Table 7.

Supplementary Table 8.

Supplementary Table 9.

Supplementary Table 10.

Supplementary Table 11.

Supplementary Table 12.

Supplementary Table 13.

Supplementary Table 14.

Supplementary Table 15.
