# Supplementary material for: Small RNAs and their targets are associated with the transgenerational effects of water-deficit stress in durum wheat
Source: Sci Rep. 2021 Feb 11;11:3613. doi: 10.1038/s41598-021-83074-7 (PMC7878867; doi:10.1038/s41598-021-83074-7)
Supplement: Supplementary file 1 — Supplementary Legends. [file 41598_2021_83074_MOESM1_ESM.docx]

**Supplementary Data**

Methods S1. Supplementary methods.

Table S1. Summary of the treatment groups and abbreviations used in the study.

Table S2. Sequencing information of the small RNA, transcriptome and degradome libraries used in this study.

Table S3. Primer sequences used in the current study.

Table S4. Summary of MIR-miRNA entries identified in this study.

Table S5. A summary of the number of DEMs (differentially expressed miRNAs) and DEGs (differentially expressed genes) subject to different factors.

Table S6. Differentially expressed miRNAs between treatment groups originating from the same parent group.

Table S7. Differentially expressed miRNAs between treatment groups with a different parent treatment factor.

Table S8. Differentially expressed miRNAs between flag leaf and developing grain tissues.

Table S9. List of genes and transcripts identified in the eight transcriptome libraries.

Table S10. Differentially expressed genes between treatment groups originating from the same parent group.

Table S11. Differentially expressed genes between treatment groups with a different parent treatment factor.

Table S12. Differentially expressed genes between flag leaf and developing grain tissues.

Table S13. Target transcripts identified via degradome sequencing in the flag leaf of DBA Artemis.

Table S14. Target transcripts identified via degradome sequencing in the developing grains of DBA Artemis.

Table S15. miRNA-mRNA pairs with significant antagonistic regulatory patterns.

Figure S1. Venn diagram showing the distribution of microRNAs across different biological groups (a) and the conservation profile of the identified durum wheat miRNAs (b). The treatment groups are: AtCC (DBA Artemis control group parents, progeny treated with control), AtCW (DBA Artemis control group parents, progeny treated with water-deficit stress), AtWC (DBA Artemis water-deficit stress group parents, progeny treated with control), AtWW (DBA Artemis water-deficit stress group parents, progeny treated with water-deficit stress). _L, denotes libraries made from the flag leaf tissue. _G, denotes libraries made from the developing grains. For reference species names: tae, *Triticum aestivum*. gma, *Glycine max*. osa, *Oryza sativa*. bdi, *Brachypodium distachyon*. ata, *Aegilops tauschii*. zma, *Zea mays*. mdm, *Malus domestica.* sbi, *Sorghum bicolor*. lus, *Linum usitatissimum*. ptc, *Populus trichocarpa*. mes, *Manihot esculenta*. vvi, *Vitis vinifera*. ath, *Arabidopsis thaliana*. cme, *Cucumis melo*. aly, *Arabidopsis lyrata*. ppe, *Prunus persica*. hvu, *Hordeum vulgare*. csi, *Citrus sinensis*. mtr, *Medicago truncatula*. cas, *Camelina sativa*. nta, *Nicotiana tabacum*. bna, *Brassica napus*. rco, *Ricinus communis*. cpa, *Carica papaya*. sly, *Solanum lycopersicum*. stu, *Solanum tuberosum*. ghr, *Gossypium hirsutum*. bra, *Brassica rapa*. sof, *Saccharum officinarum*. ssp, *Saccharum ssp.* gra, *Gossypium raimondii*. hbr, *Hevea brasiliensis*. lja, *Lotus japonicas*. far, *Festuca arundinacea*. aqc, *Aquilegia caerulea*. cca, *Cynara cardunculus*. rgl, *Rehmannia glutinosa*. ttu, *Triticum turgidum*.

Figure S2. qPCR analysis of eight stress-responsive miRNAs in the flag leaf tissue (denoted with _L, highlighted in blue) and in the developing grains (denoted with _G, highlighted in orange) of DBA Aurora. Relative miRNA expression was calculated using GAPDH as the housekeeping gene. Data are presented as the mean ± standard error (SE) (n = 3). One-way ANOVA was used to determine statistical significance across treatment groups at *P* < 0.05 with the l.s.d. value (least significant difference). Different letters (a – c) denote the statistical difference across the treatment groups. The treatment groups are: AuCC (DBA Aurora control group parents, progeny treated with control), AuCW (DBA Aurora control group parents, progeny treated with water-deficit stress), AuWC (DBA Aurora water-deficit stress group parents, progeny treated with control), AuWW (DBA Aurora water-deficit stress group parents, progeny treated with water-deficit stress).

Figure S3. qPCR analysis of 12 stress-responsive target genes in the flag leaf tissue (denoted with _L, highlighted in blue) and in the developing grains (denoted with _G, highlighted in orange) of DBA Aurora. The miRNA targeting the gene is shown in brackets. Relative gene expression was calculated using GAPDH as the housekeeping gene. Data are represented as the mean ± standard error (SE) (n = 3). One-way ANOVA was used to determine statistical significance across treatment groups at *P* < 0.05 with the l.s.d. value (least significant difference). Different letters (a – c) denote the statistical difference across the treatment groups. The treatment groups are: AuCC (DBA Aurora control group parents, progeny treated with control), AuCW (DBA Aurora control group parents, progeny treated with water-deficit stress), AuWC (DBA Aurora water-deficit stress group parents, progeny treated with control), AuWW (DBA Aurora water-deficit stress group parents, progeny treated with water-deficit stress). SRO1, poly [ADP-ribose] polymerase SRO1 gene.
