# Supplementary figures and images for: Small RNAs and their targets are associated with the transgenerational effects of water-deficit stress in durum wheat
Source: Sci Rep. 2021 Feb 11;11:3613. doi: 10.1038/s41598-021-83074-7 (PMC7878867; doi:10.1038/s41598-021-83074-7)

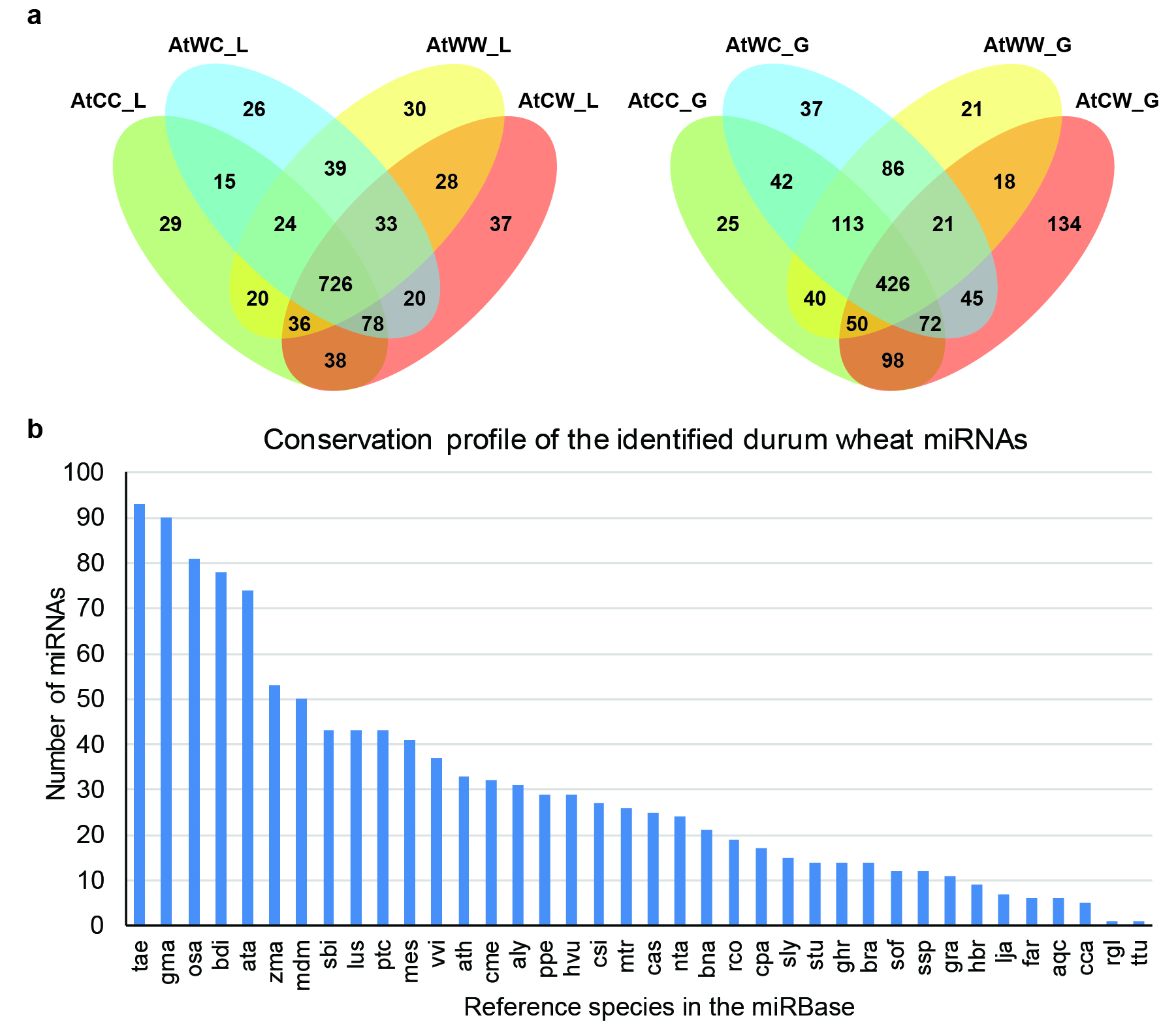

Supplement: Supplementary file 2 — Supplementary Figure 1. [file 41598_2021_83074_MOESM2_ESM.jpg]

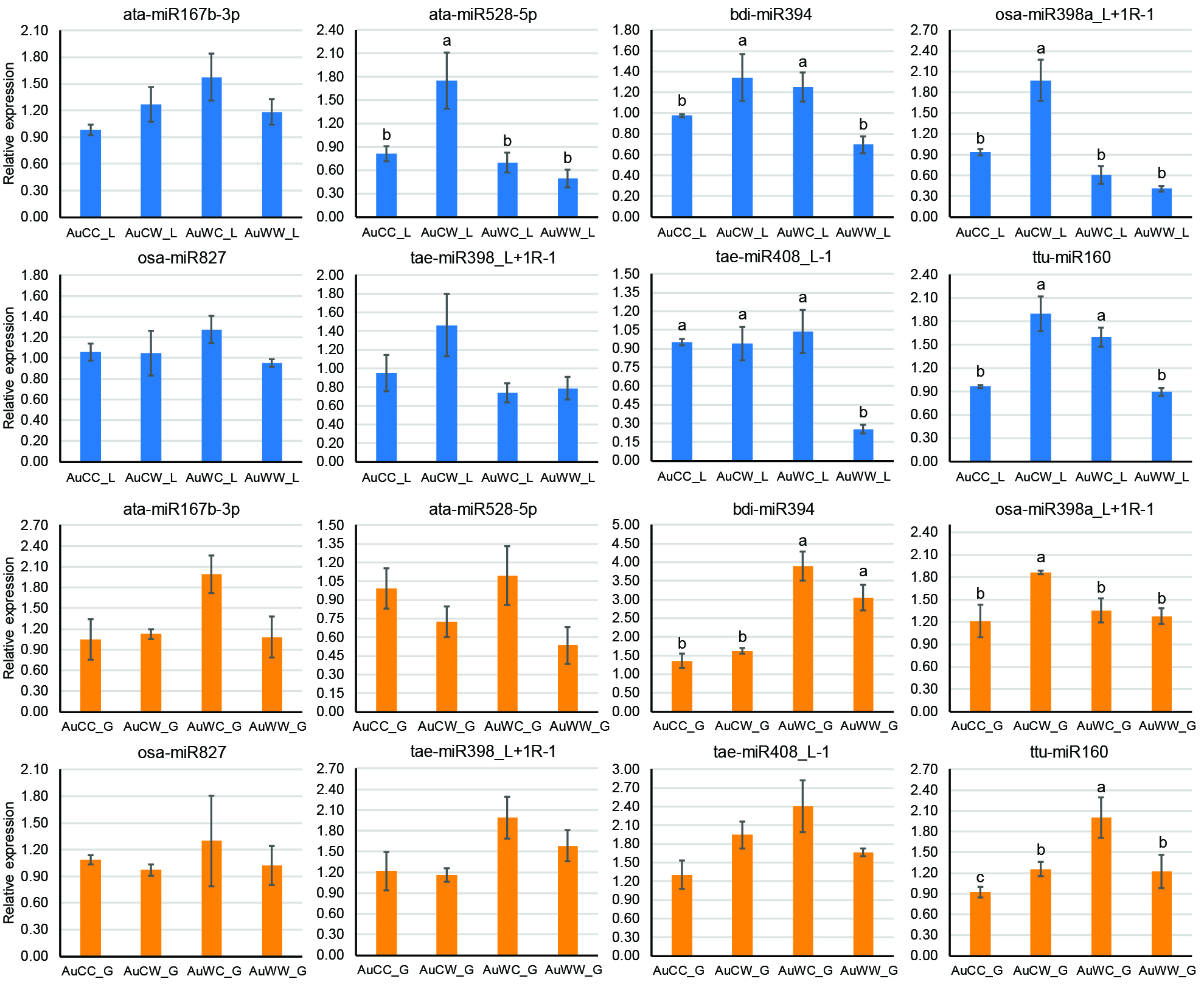

Supplement: Supplementary file 3 — Supplementary Figure 2. [file 41598_2021_83074_MOESM3_ESM.jpg]

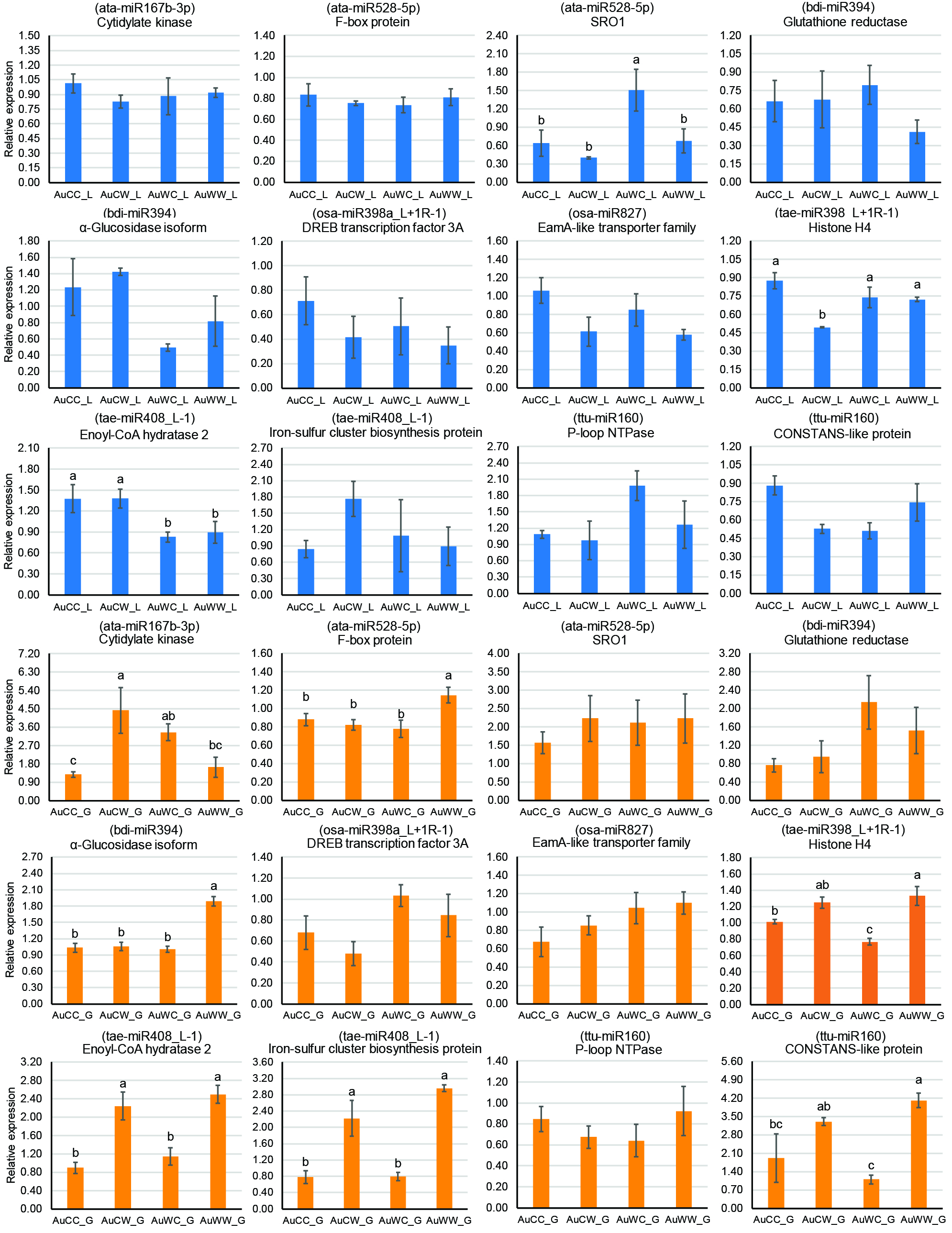

Supplement: Supplementary file 4 — Supplementary Figure 3. [file 41598_2021_83074_MOESM4_ESM.jpg]
