# Supplementary material for: Small RNAs and their targets are associated with the transgenerational effects of water-deficit stress in durum wheat
Source: Sci Rep. 2021 Feb 11;11:3613. doi: 10.1038/s41598-021-83074-7 (PMC7878867; doi:10.1038/s41598-021-83074-7)
Supplement: Supplementary file 5 — Supplementary Information. [file 41598_2021_83074_MOESM5_ESM.docx]

**Small RNAs and their targets are associated with the transgenerational effects of water-deficit stress in durum wheat**

Haipei Liu^1*^, Amanda J. Able^1^, Jason A. Able^1^

^1^ School of Agriculture, Food & Wine, Waite Research Institute, The University of Adelaide, Urrbrae, SA 5064, Australia

**Methods S1.** Supplementary information on the experimental procedures.

**Small RNA sequencing analysis**

Small RNA sequencing analysis was performed using the ACGT101-miR program (LC Sciences, USA) as previously described ^1-3^. Raw reads were first processed by removing low-quality reads and adapter sequences to obtain clean sequences. Reads with a nucleotide (nt) length <18 nt or >25 nt were removed. Non-coding (nc) RNA families (rRNA, tRNA, snRNA and snoRNA), repeats and mRNA sequences were discarded using RFam, Repbase and durum wheat NCBI mRNA entries as references. Unique sRNA sequences were then obtained for each library. Conserved mature miRNAs and 5p- or 3p-derived miRNA variants were identified using BLAST search against the plant miRBase (Release 22.1). MIR (microRNA gene locus) and miRNA sequences from common plant species in the miRBase were used as references. Sequences mapped to the mature miRNA in the hairpin were identified as conserved mature miRNAs. Sequences mapped to the opposite arm of mature miRNA in the hairpin were identified as 5p- or 3p-derived variants. Single mismatch within the sequence and length variation at both 5p and 3p were allowed in the alignment. All the mapped miRNAs were aligned to the durum wheat genome (NCBI UID 3439611, assembly Svevo.v1) to determine their genomic location. The remaining unmatched sRNA sequences were used to identify novel durum miRNAs. Sequences were BLASTed to the durum wheat genome. To identify miRNA precursors, secondary hairpin structures containing matched sequences were predicted using RNAfold (http://rna.tbi.univie.ac.at/cgi-bin/RNAfold.cgi) from 120nt of flanking genome sequences as previously described ^1^. All miRNAs were categorised into five groups (G1-5), where group 1 to group 4 represent conserved miRNAs, while group 5 represents novel miRNAs ^1^. The definitions of each group are as follows: G1, reads can be mapped to miRNAs in the miRBase, and the pre-miRNA can be mapped to the durum wheat genome; G2, reads can be mapped to miRNAs in the miRBase, but the pre-miRNA cannot be mapped to the durum wheat genome. However the reads can be mapped to the genome, and the extended genome sequences from the mapped location can form secondary hairpins; G3, reads can be mapped to miRNAs in the miRBase, but the pre-miRNA cannot be mapped to the durum wheat genome. However the reads can be mapped to the genome, and the extended genome sequences from the mapped location cannot form secondary hairpins; G4, reads can be mapped to miRNAs in the miRBase, either the pre-miRNA or reads can be mapped to the durum wheat genome; G5, reads cannot be mapped to the miRBase, but can be mapped to the durum wheat genome. Secondary hairpins can be formed from extended genome locations.

**Transcriptome sequencing analysis**

For transcriptome-seq analysis, low quality reads (those containing primer or adaptor sequence, and those with a sequencing quality score < 20) were removed prior to sequence assembly. Clean reads were aligned to the durum reference genome using the HISAT package (V2.0). Aligned reads were assembled and the transcript abundance were obtained using StringTie (V1.3.0) for each transcriptome library. Normalised relative abundance for each gene was expressed in FPKM (Fragments Per Kilobase Million) for each library.

**Degradome sequencing analysis**

Around 20µg of total RNA per sample was used for degradome-seq library construction ^3,4^. First, mRNA was enriched using oligo-d(T) magnetic beads. The enriched mRNA was mixed with biotinylated random primers and were ligated to 5' adaptors. First-strand cDNA was reverse-transcribed from ligated mRNA products and then amplified with PCR. The synthesised cDNA libraries were sequenced on an Illumina Hiseq2500 (LC-BIO, Hangzhou, China). Raw sequencing reads were processed using the ACGT101-DEG program (V4.1, LC Sciences, USA) to remove low-quality reads, reads with adaptor and primer contamination, and reads that can be annotated as non-coding RNA families. The remaining clean reads were used to identify the degraded fragments of mRNAs that are targets of known and novel durum miRNAs with the CleaveLand package V4.0 (Addo-Quaye et al., 2009a,b) and the ACGT101-DEG program (LC Sciences, TX, USA). The mRNA cleavage sites at the 10th position of miRNA alignment with *P* value < 0.05 were considered as significant.

**Supplemental References**

1 Liu, H., Able, A. J. & Able, J. A. Transgenerational effects of water-deficit and heat stress on germination and seedling vigour - new insights from durum wheat microRNAs. *Plants* **9**, 189 (2020).

2 Liu, H., Able, A. J. & Able, J. A. Multi-omics analysis of small RNA, transcriptome, and degradome in *T. turgidum* - regulatory networks of grain development and abiotic stress response. *Int. J. Mol.* **21**, 7772 (2020).

3 Liu, H., Able, A. J. & Able, J. A. Integrated analysis of small RNA, transcriptome and degradome sequencing reveals the water-deficit and heat stress response network in durum wheat. *Int. J. Mol.* **21**; 10.3390/ijms21176017 (2020).

4 Zhong, M. *et al.* The effect of cadmium on the microRNAome, degradome and transcriptome of rice seedlings. *Plant Growth Regul.* **90**, 15-27 (2020).
